# Chromatin Nano-Organization in Peripheral Blood Mononuclear Cells After In-Solution Irradiation with the Beta-Emitter Lu-177

**DOI:** 10.3390/biom16010142

**Published:** 2026-01-13

**Authors:** Myriam Schäfer, Razan Muhtadi, Sarah Schumann, Felix Bestvater, Uta Eberlein, Georg Hildenbrand, Harry Scherthan, Michael Hausmann

**Affiliations:** 1 Kirchhoff-Institute for Physics, Heidelberg University, Im Neuenheimer Feld 227, 69120 Heidelberg, Germany; myriam.schaefer@kip.uni-heidelberg.de (M.S.); georg.hildenbrand@th-ab.de (G.H.); 2 Bundeswehr Institute of Radiobiology affiliated to the University of Ulm, Neuherbergstraße 11, 80937 München, Germany; razan1muhtadi@bundeswehr.org; 3 Department of Nuclear Medicine, University of Würzburg, Oberdürrbacher Str. 6, 97080 Würzburg, Germany; schumann_s1@ukw.de (S.S.); eberlein_u@ukw.de (U.E.); 4 German Cancer Research Center (DKFZ), Im Neuenheimer Feld 280, 69120 Heidelberg, Germany; f.bestvater@dekfz-heidelberg.de; 5 Faculty of Engineering, University of Applied Sciences Aschaffenburg, Würzburger Str. 45, 63743 Aschaffenburg, Germany

**Keywords:** low-LET β-irradiation, Lu-177 radioactive decay, nanoscale spatial organization of γH2AX, 53BP1, MRE11, H3K9me3, single-molecule localization microscopy, Ripley statistics, persistent homology, persistent imaging

## Abstract

**Background**: In nuclear medicine, numerous cancer types are treated via internal irradiation with radiopharmaceuticals, including low-LET (linear energy transfer) beta-emitting radionuclides like Lu-177. In most cases, such treatments lead to low-dose exposure of organ systems with β-irradiation, which induces only few isolated DSBs (double-strand breaks) in the nuclei of hit cells, the most threatening DNA damage type. That damaging effect contrasts with the clustering of DNA damage and DSBs in nuclei traversed by high-LET particles (α particles, ions, etc.). **Methods**: After in-solution β-irradiation for 1 h with Lu-177 leading to an absorbed dose of about 100 mGy, we investigated the spatial nano-organization of chromatin at DSB damage sites, of repair proteins and of heterochromatin marks via single-molecule localization microscopy (SMLM) in PBMCs. For evaluation, mathematical approaches were used (Ripley distance frequency statistics, DBScan clustering, persistent homology and similarity measurements). **Results**: We analyzed, at the nanoscale, the distribution of the DNA damage response (DDR) proteins γH2AX, 53BP1, MRE11 and pATM in the chromatin regions surrounding a DSB. Furthermore, local changes in spatial H3K9me3 heterochromatin organization were analyzed relative to γH2AX distribution. SMLM measurements of the different fluorescent molecule tags revealed characteristic clustering of the DDR markers around one or two damage foci per PBMC cell nucleus. Ripley distance histograms suggested the concentration of MRE11 molecules inside γH2AX-clusters, while 53BP1 was present throughout the entire γH2AX clusters. Persistent homology comparisons for 53BP1, MRE11 and γH2AX by Jaccard index calculation revealed significant topological similarities for each of these markers. Since the heterochromatin organization of cell nuclei determines the identity of cell nuclei and correlates to genome activity, it also influences DNA repair. Therefore, the histone H3 tri methyl mark H3K9me3 was analyzed for its topology. In contrast to typical results obtained through photon irradiation, where γH2AX and H3K9me3 markers were well separated, the results obtained here also showed a close spatial proximity (“co-localization”) in many cases (minimum distance of markers = marker size), even with the strictest co-localization distance threshold (20 nm) for γH2AX and H3K9me3. The data support the results from the literature where only one DSB induced by low-dose low LET irradiation (<100 mGy) can remain without heterochromatin relaxation for subsequent repair.

## 1. Introduction

The last decade has seen growing application of radiopharmaceutical cancer treatments involving internal irradiation with β-emitters like Lu-177 (lutetium 177) [1,2]. Lu-177 is used for both imaging and treatment, especially in the theranostics of metastatic prostate and gastric cancers [3]. Since most radiopharmaceuticals involve beta-emitters that are usually administered via intravenous injection, the blood of the patients is also exposed to low doses of this sparsely ionizing radiation during therapy, leading to scattered DSB damage in peripheral blood mononuclear cells (PBMCs). A major risk factor of cancer treatment using ionizing radiation is the potential exposure of the radiosensitive bone marrow and other healthy tissues, which may induce a decrease in erythrocyte and PBMC counts as side effects [4,5,6,7]. Since peripheral blood is an easily obtainable tissue and a surrogate marker for the radiosensitive bone marrow, there has been research on PBMCs regarding the relationship between the absorbed dose and radiation-induced DNA double-strand break (DSB) foci containing γH2AX+53BP1 simultaneously [8]. The most threatening DNA damage type is the DSB, which triggers a DNA damage response (DDR) in the hit cells that may lead to DNA repair or to cell death. The latter is the favorable aim in cancerous cells and determines the effectiveness of tumor treatment [9,10].

Ionizing radiation (IR)-induced DNA damage leads to the activation of specific repair mechanisms that attempt to maintain the integrity of the DNA molecule [11]. The prevalent DSB repair pathways are non-homologous end joining (NHEJ) [12] and homologous recombination repair (HRR) [13]. Although rapid DNA repair via NHEJ is in principle error-prone, it predominates in euchromatin during the entire cell cycle [14,15]. HRR, on the other hand, requires an intact DNA sequence template on the homologous chromosome and is generally only active in late S and G2 and occurs preferentially in heterochromatin [14,16,17,18]. If NHEJ fails, for example, in mutant situations and at doses exceeding 8 Gy, an alternative NHEJ process (a-NHEJ) may be initiated, which is slower and generally error-prone [19,20].

DNA in the nucleus is present as a histone protein/DNA complex termed chromatin, the local organization of which impacts DSB repair pathways whose protein components attach to the chromatin at the DNA damage site in a given sequence. While low-dose irradiation with low-energy transfer (LET) β-emitters or photon irradiation will induce only few DSBs per nucleus, high-LET particulate irradiation with α particles or accelerated ions induces dense ionizations along particle tracks that induce significant chromatin alterations and accumulation of DNA damage types modulating DNA repair and 3D genome organization [21,22,23].

Here, we focus on the spatial organization of the following DSB repair-associated proteins that accumulate around isolated DSBs induced in PBMC nuclei by low-LET, low-dose β-irradiation [24].

pATM is the form of the PI3-related kinase ATM phosphorylated at S 1981. After the induction of DSBs, the MRN complex binds to the break ends and activates ATM [25]. Inactive ATM is present in cells as a dimer or multimer. Autophosphorylation of ATM upon DNA damage causes dissociation of these dimers, releasing active ATM monomers [26]. ATM phosphorylates itself and several key proteins that trigger the DDR, leading to cell cycle arrest, DNA repair or apoptosis [27]. The ATM kinase is, furthermore, crucial for extensive histone H2AX phosphorylation around irradiation-induced DSBs [28]. It is also thought that pATM is involved in DNA damage detection and may serve as a sensor in relation to radiation sensitivity [29,30,31,32].

γH2AX is formed seconds after DSB formation by phosphorylation of the histone H2AX throughout a one- to two-megabase region around a DSB [33,34,35,36,37,38], which ensures the recruitment of various dsDNA damage responsive proteins, such as NSB1, 53BP1, BRCA1, MRN, etc. [36,37]. Microscopically visible γH2AX macro foci are a sensitive surrogate marker of DSBs and can be detected as early as 20 s after cell irradiation and are part of the early DDR after heterochromatin decondensation [36,38].

53BP1 is one of the early DDR proteins, binding to the chromatin environment of a DSB shortly after γH2AX formation. 53BP1 plays a central role in tumor suppression and regulation of DSB repair signaling [39,40]. As an important mediator in checkpoint signaling, 53BP1 can enhance the kinase activity of ATM and increase the accumulation of BRCA1 at the break sites during the G1-phase, preferentially initiating NHEJ [41,42].

MRE11 (Meiotic recombination 11) together with RAD50 and NBS1 is part of the MRN complex [43,44,45,46], which detects DSBs and triggers the DDR by activating the ATM kinase [47,48]. MRE11 is involved in both HRR and NHEJ repair mechanisms due to its 3′-5′ DNA endonuclease and exonuclease activity [49]. During HRR, the nuclease generates the 3′ single-stranded overhang of DNA at the break ends. In NHEJ, MRE11 plays an important role in DNA processing. Like 53BP1, MRE11 also forms foci at the damage sites, which in turn can co-localize with γH2AX foci. It has been hypothesized that MRE11 is also involved in checkpoint signaling [48,49]. In contrast to 53BP1, which binds over the complete γH2AX region [23], MRE11 appears to be integrated at or between the broken ends of a DSB [50,51]. Furthermore, a different time course of MRE11’s presence at DSBs was shown for fibroblasts in comparison to MCF7 breast cancer cells [50].

It has been recognized that chromatin compaction influences the processing of DSBs and their repair [52]. Heterochromatin is a compacted and genetically silent form of chromatin that depends on the presence of methyl chromatin marks on histones [53]. The tri-methylated form of histone H3 Lysin 9 (H3K9me3) is highly concentrated in constitutive heterochromatin of silent gene regions, such as centromeres or telomeres. Trimethylation of H3K9 in the promoter region of genes is thought to prevent their overexpression and act as a transcriptional repressor [53]. For example, H3K9me3 can induce the p53-dependent apoptosis signaling pathway by inhibiting the expression of the regulator protein APAK [54]. Methylation of lysine-9 of H3 contributes to cell identity determination [55]. It may also play an important role in ATM kinase activation [55]. Currently, the extent to which this methylation is regulated is under investigation. Furthermore, accumulation of H3K9me3 has been used as a marker for the condensation status of heterochromatin in pathological conditions [56].

Chromatin organization has been shown to interact with genetic activity [57], to respond to ionizing radiation and other genotoxic exposures [58,59], and is known to influence DNA repair. The DDR differently correlates with changes in heterochromatin reorganization depending on the cell type and the type and dose of ionizing radiation [60].

Chromatin compaction has been shown to influence DNA repair fidelity [55]. While low-dose low-LET irradiation usually results in well-spaced single DSBs distributed throughout the cell nuclei, high-LET particle irradiation results in ionization tracks traversing the chromatin along which complex chromatin damages and consequently DDR proteins accumulate [23]. Thus, for low-LET irradiation, the number of γH2AX foci per cell can be used as an absorbed-dose measure in biological dosimetry [61], whereas this is not possible for high-LET radiation with complex accumulations of DNA damages and γH2AX foci within the tracks [62]. Previously, PBMCs of blood samples of six individuals were internally irradiated with the α-particle emitter Ra-223 (high-LET) at low doses (<150 mGy). For these cases, it was shown that the number of tracks is a relatively good measure for the dose absorbed by the cells [63]. Choosing those cells with typically one α-track per nucleus, the analysis of these DNA damage tracks via single-molecule localization microscopy (SMLM) showed the characteristics of γH2AX, 53BP1 cluster formation, and the localization of the single-molecule distribution of MRE11 and pATM within these tracks [23]. SMLM [64,65] appears to be a powerful tool to investigate the properties of irradiation tracks and their repair protein organization at sub Abbé limit resolution in more detail and, thus, to gain a better understanding of DNA damage processing in such cells [66]. Here, we extend our Ra-223 investigation [23] and study the distribution of DDR proteins at DSB chromatin regions around simple DSBs induced by ex vivo internal low-dose β-irradiation with Lu-177 of peripheral blood mononuclear cells [24,67].

## 2. Materials and Methods

### 2.1. Cell Preparation, Irradiation and Immuno-Labeling

The white blood cells from internally Lu-177-irradiated peripheral blood samples were prepared as described in detail elsewhere. In short, blood samples were collected from a healthy anonymous donor, divided into 3.5 mL aliquots and mixed with 1 mL radioactive solution of 0.9% NaCl and Lu-177 with known activity, corresponding to a dose rate of 100 mGy/h [24,67]. To ensure a homogenous distribution of the activity added to the blood sample, we incubated the sample for one hour at 37 °C on a roller mixer (35 rpm). To verify that the activity concentration in the blood after the incubation time corresponds to the pre-calculated value, a test sample of each irradiated sample was measured in a calibrated germanium detector. Given that the irradiation was homogenous under these conditions, we assumed that the absorbed dose was the same throughout the entire sample volume, i.e., 100 mGy. In contrast to the high linear energy-transmitting α particles that have a path length of <0.1 mm and deposit energies in the range of several MeV (e.g., 5.77 MeV for Ra-223), the low-LET Lu-177-emitted β-particles have a maximum energy of 496.8 keV (http://www.lnhb.fr/home/nuclear-data/nuclear-data-table/, accessed 12 November 2025) and a path length up to 2 mm (https://physics.nist.gov/PhysRefData/Star/Text/ESTAR.html, accessed 12 November 2025).

After internal irradiation, PBMCs were isolated from the Lu-177-containing blood solution via density centrifugation in CPT Vacutainer tubes (1500× *g*) for 20 min, extracted above a separation layer and washed twice with phosphate-buffered saline (PBS). The cell suspension was fixed with ice-cold 70% ethanol. Samples were stored at −20 °C until immunofluorescence staining, as described [68]. In brief, the cells fixed in ethanol were subjected to cyto-centrifugation followed by immunofluorescent staining. The following primary antibodies, mouse anti-γH2AX (Merck Chemicals, Darmstadt, Germany), rabbit anti-MRE11 (Novus, Abingdon, UK), rabbit anti-phospho-ATM (pS1981; abcam, Cambridge, UK), and rabbit anti-53BP1 (Novus, Abingdon, UK), were applied in TCTG buffer (Tris-HCl, 0. 1% casein, 0.05% Tween-20, 0.3% fish gelatin) and then incubated overnight at 4 °C. After washing three times for 5 min in TCTG at 37 °C, the samples were incubated with the secondary antibodies (anti-mouse-Cy3 and anti-rabbit-Cy5) for 45 min at 37 °C (both Dianova, Hamburg, GER). Finally, the cells were washed five times for 3 min in TCTG buffer at 37 °C and embedded in Prolong Gold antifade solution (Life Technologies, Carlsbad, CA, USA) containing 4′,6- diamidino-2-phenylindole (DAPI) as DNA-specific counterstain [49].

### 2.2. Single-Molecule Localization Microscopy (SMLM)

SMLM [64,65] is based on the concept that switching fluorescence between two different spectral excitation states, blinking can be achieved, resulting in a temporal and spatial separation of molecular signals. The microscopic setup is described in detail in [23,51].

We applied a motorized inverted TILL Photonics FEI microscope equipped with four lasers (excitation wavelength/maximum power: 405 nm/120 mW; 491 nm/200 mW; 561 nm/200 mW; 642 nm/140 mW (not used in this study). The DAPI counter-staining was excited with a 405 nm laser in order to visualize the margins of the nuclei; the 642 nm laser was used for γH2AX (Cy3-marked) excitation and the 561 nm laser for all Cy5-marked proteins (53BP1, MRE11, pATM, H3K9me3). We adjusted excitation laser wavelength and intensity using a polychromatic AOTF (AA Opto Electronic, Orsay Cedex, France). A variable beam expander 10BE03-2-8 (Standa Ltd., Vilnius, Lithuania) and a Flat-Top-Profile forming optics PiShaper (AdlOptica GmbH, Berlin, Germany) expanded and homogenized the laser beam. The circular Flat-Top laser beam profile was projected into the object plane by an achromatic focusing lens (f = 250 mm) and a 100x/NA 1.46 oil plan apochromatic objective lens (Carl Zeiss Microscopy, Göttingen, Germany). Emission and excitation paths were separated by two quadband interference filter glasses F73-410 and F72-866 (AHF Analysentechnik AG, Tübingen, Germany). Objective and tube lenses (Carl Zeiss Microscopy, Göttingen, Germany) and an additional twofold expander further projected (magnified) the emission light. An iXon Ultra Andor electron-multiplying charge-coupled device (EMCCD) camera (Andor Technology, Belfast, UK) was used to record the SMLM signals.

Granulocytes were excluded because of their polymorphic shape, and only intact nuclei that show foci in both channels were taken into account. During measurement, each nucleus was flashed with a laser intensity of 70% (note that the optical components like the AOTF have an impact on the real laser power at the object slide) for 3000 ms to reduce background signals, following the acquisition of 2000 images using an EM-gain of 100 and an exposure time of 100 ms per frame. For each slide, on average, 25 nuclei with a typical size of about 30 µm^2^ were considered for the evaluation of damage sites. It should be mentioned that, in principle, more images (up to 10,000) can be recorded. Since we are not analyzing images but the coordinates in the orte-matrix, systematic studies in our lab revealed that about 2000 images are enough for obtaining the full information.

### 2.3. SMLM Data Evaluation

The evaluation of these raw image stacks was carried out with various in-house prepared analysis programs [69]. In brief, two masking methods were applied for each nucleus: (a) based on the DAPI labelling (overview image), the first mask excluded all signals outside the cell nucleus, and (b) the manually processed foci mask using the γH2AX overview image considered only signals inside the relevant DSB regions. Then, a two-dimensional Gaussian fit (threshold factor = 3) was applied to determine the position of each detected fluorophore (blinking event) [70]. The resulting coordinate data of the labeling points were summarized in a so-called “orte matrix” containing signal amplitudes, lateral x- and y-coordinates and localization errors of each sample. The localization precisions were 17 nm for Cy3 and 15 nm for Cy5 labelling.

Further analyses using DBscan (Density-Based Spatial Clustering of Applications with Noise) [71] and Ripley’s k-statistic were performed to obtain information about the pairwise distances between the detected events and the accumulation in clusters. A value of 200 nm was chosen for the cluster radius and 30 counts for the minimum number of signals inside a cluster. For the co-localization criteria, a distance limit of 95 nm between labeling signals in the first channel to the nearest signal in the second channel was selected, assuming the space of the antibody construction required for stoichiometric reasons (detailed calculations are described in [72]).

The parameters chosen are consistent with those used in previous experiments [23]. Lastly, persistent homology analysis [73,74] was performed to compare the topology of the protein clusters and to investigate whether they contain similar, recurring structures or substructures. This was achieved by filtering the coordinates of the detected signals using the α-shape method, whereas α is a gradually increasing radius of an imaginary circle around every point inside the nucleus or a cluster. The similarity between the components or holes in point clouds (protein distribution) was assessed using the Jaccard index [75], determining the overlap of one barcode [76] with another and vice versa. The Jaccard index is the normalized measure for similarity between finite non-empty sample sets and is defined as the size of the intersection divided by the size of the union of the sample sets. By design, it ranges from no similarity = 0 ≤ J (A,B) ≤ 1 = identity. If sets A and B have no elements in common, their intersection is empty, so |A ∩ B| = 0 and therefore J (A,B) = 0. The other extreme is that the two sets are equal. In that case, A ∩ B = A ∪ B = A = B so then J (A,B) = 1. The Jaccard index is widely used in computer science, ecology, genomics and other sciences where binary or binarized data are used. The Jaccard indices of one labeling type were summarized in a heatmap of the 1st generation each. Finally, the average similarities obtained as the mean values of the single 1st-generation heatmaps were transferred in a heatmap of the 2nd generation [50], which allowed for the comparison of multiple datasets.

## 3. Results

**Figure 1:**
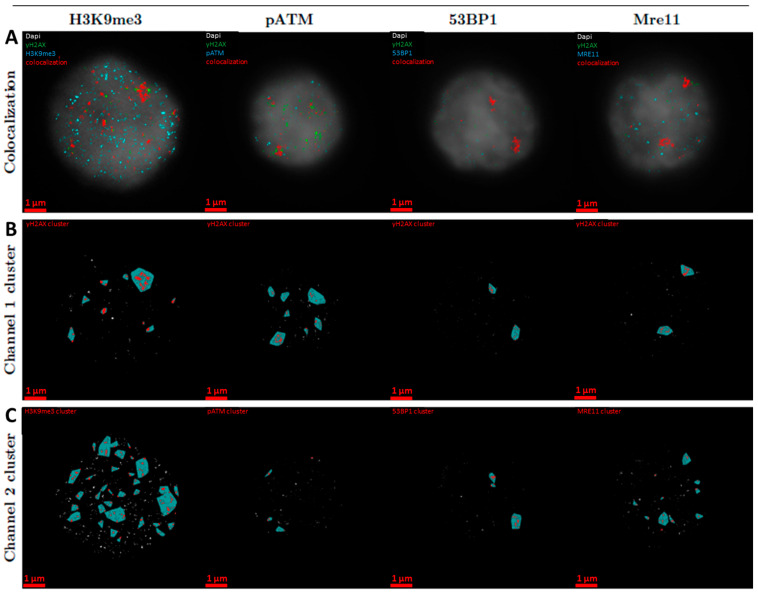
Typical SMLM images of the distribution of different repair protein signal tags in PBMNC nuclei exposed to β-radiation: (**A**) In each image γH2AX signals are visualized in green, the signals of the second label (H3K9me3, pATM, 53BP1, or MRE11) are shown as light blue dots and the co-localization of both labels (distance between signal tagsof two different channels < 95 nm) as red dots. (**B**,**C**) Cluster images for each label pair (**B**) γH2AX; (**C**) H3K9me3, pATM, 53BP1 or MRE11. Green-blue areas represent the convex hull of the clusters with the red dots obtained by DBscan (N_min_ = 30, R = 200 nm), while white dots represent the signals outside the cluster. Scale-bar: 1 µm.

Figure 1 shows typical examples of PBMC cell nuclei after in-solution Lu-177 β-particle irradiation. Since the cell nuclei were randomly hit by the low-dose β-particle irradiation, typically one or two DSB foci per nucleus were obtained at the 100mGy dose absorbed over 1 h showing focal signals for γH2AX, pATM, 53BP1, or MRE11. As expected, the heterochromatin mark H3K9me3 was prominent at the nuclear periphery. In Figure 1A, the DAPI image of the background is visible. As expected, the clusters of pATM, 53BP1 and MRE11 locate in the γH2AX-outlined chromatin regions.

**Figure 2:**
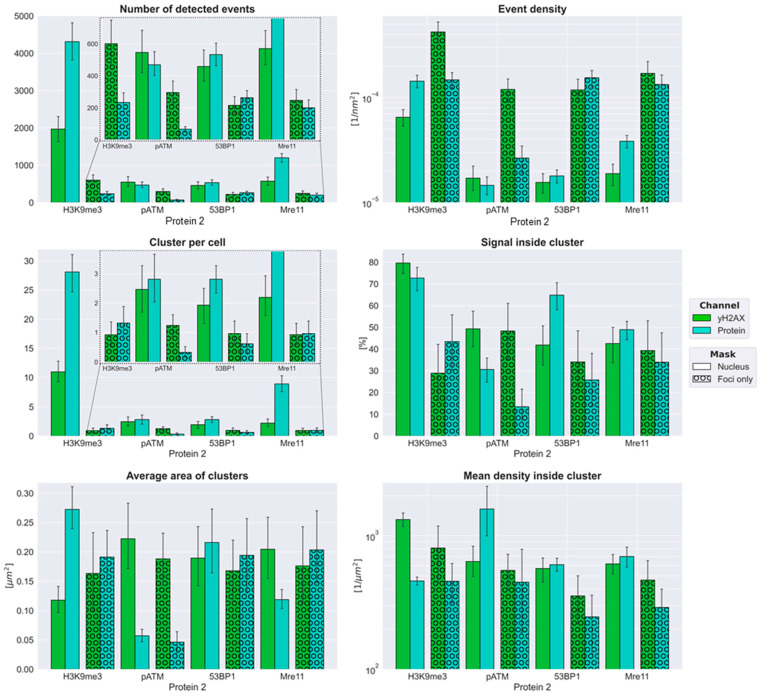
SMLM values of detected events and cluster properties are shown for the entire cell nucleus (defined by the DAPI mask) and the masked areas defined by γH2AX macro foci. For each experiment, the respective γH2AX values are presented together with those of a second protein. For instance, in the “number of detected events” histogram labelled H3K9me3, the four columns represent: (1) γH2AX tags in the whole nucleus, (2) H3K9me3 tags in the whole nucleus, (3) γH2AX tags within γH2AX foci masks, (4) H3K9me3 tags within γH2AX foci masks. The same layout applies when H3K9me3 is replaced by pATM, 53BP1, or MRE11. From top left to bottom right, the panels show: number of detected events (tags), mean event density (tags/µm^2^), mean number of clusters, mean number of tags inside clusters, mean cluster area, and mean tag density within clusters.

For each protein, the position of each signal tag was determined by SMLM, and the number of blinking events and their event density were calculated. Furthermore, the clustering behavior of the proteins at the sub-Abbé-limit was studied in more detail (Figure 2).

For each secondary label (H3K9me3, pATM, 53BP1, or MRE11), four columns are shown: mean value of γH2AX single-molecule tags in the masked nuclei (DAPI staining), mean value of the second labels in the masked nuclei (DAPI staining), mean value of γH2AX tags in the masked γH2AX foci, and mean values of the second label in the masked γH2AX foci (typically about 6% of a nucleus) separately. Of all the DDR proteins, MRE11 had the highest tag numbers in the cell nuclei, more than twice as frequently as γH2AX tags. 53BP1 signal tags were also more abundant than γH2AX. Up to 80% of the γH2AX tags were found in clusters considering the whole nucleus. Considering the γH2AX mask only, about 40% of the 53BP1 tags were found in clusters. This was in agreement with the result obtained in experiments on γ-irradiated HeLa cells, where a ratio of 0.1–0.5 tags within clusters relative to the total amount of tags in the nucleus was estimated [35]. A higher amount of 53BP1 signals in clusters compared to γH2AX was also compatible with the findings of previous studies on heavy ion irradiation of fibroblast and glioblastoma cells [77,78].

53BP1 and MRE11 signal tags were randomly distributed over the nucleus, when they were not present in the chromatin near DSB damage. The latter regions were marked by the radiation-induced phospho proteins γH2AX and pATM, with γH2AX histone phosphorylation taking place in proximity (~1 MB) to the DSB. Despite the larger number of clusters, MRE11 showed lower signal values within the clusters, as compared to 53BP1. The fluorophore number of pATM signal tags per nucleus was slightly lower than that of γH2AX. This has also been observed in previous investigations [23] and could be a result of a temporally limited presence of the activated ATM kinase in the DSB region.

H3K9me3 showed the largest cluster size of about 0.27 µm^2^, more than twice the size of the γH2AX clusters detected in nuclei of the same slide. The values of the determined cluster areas of γH2AX and 53BP1 varied between 0.12 and 0.23 μm^2^, which is consistent with values in the literature [38].

Compared to its distribution over a nucleus (smaller cluster sizes compared to γH2AX), MRE11 formed slightly larger protein clusters than γH2AX in DSB foci regions. Similarly, after applying the foci mask, the number of MRE11 clusters in the mask had converged with those of the γH2AX values. The number of MRE11 clusters was equal to the number of γH2AX clusters in DSB foci, whereas a significantly higher cluster number for MRE11 was detected throughout the nucleus without γH2AX co-localization. This is likely due to the fact that MRE11 (respectively, the MRN complex) is permanently present in the cell nucleus [79], controlling chromatin integrity and other nuclear functions. pATM had the lowest numbers of signal tags and clusters but showed a compatible density to γH2AX within γH2AX macro-foci. In the cell nucleus, the mean density of pATM in nano-clusters was higher than γH2AX, which, however, was due to the smaller cluster size. 53BP1 shows about the same signal density as γH2AX but a slightly increased cluster area, which is compatible with former observations [23,49].

**Figure 3:**
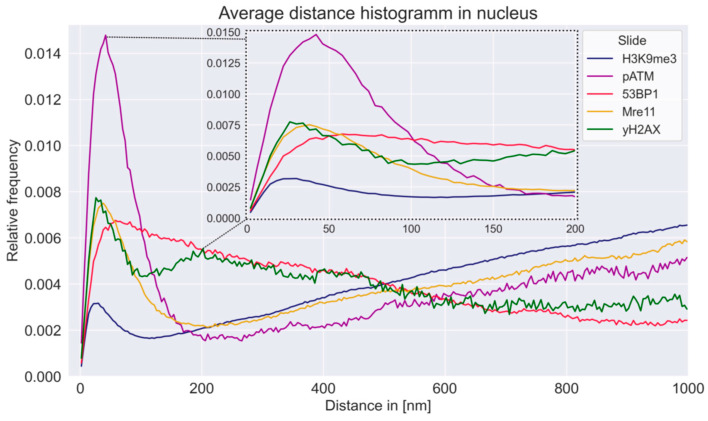
Ripley histograms showing relative pair-wise point distance frequencies for all labeling tags. Peaks below ~200 nm indicate cluster formation, while the continuous increase at larger distances reflects random tag random tag distribution. The insert highlights the peaks at a higher distance resolution.

The distances between detected fluorophore single-molecule tags were calculated according to Ripley’s k-statistic [80] and plotted in histograms describing relative abundance as a function of point-to-point distance (Figure 3). These curves provided more detailed insight into the distribution of each repair protein inside a nucleus. In these curves, non-random distributions due to clustering appear as individual peaks on the left (small point-to-point distances). The maximum value of a peak represents the characteristic spacing of a cluster. The symmetry and width of the individual peaks reflect the spatial organization of the markers. The distribution of spatially disordered background signals resembled a monotonically increasing linear function. A plateau or broader secondary peak at 200 nm was observed for the γH2AX signals in the nucleus, suggesting that the clusters were in close proximity to other γH2AX assemblies (foci substructures) or that smaller and larger cluster types occurred. The distance from one peak to the other was about 170 nm.

The peak of MRE11 well fits with the first γH2AX peak, while the peak of 53BP1 is broader and overlapping the first γH2AX peak. Two adjacent γH2AX peaks, however, do not require two peaks for the repair proteins MRE11 and 53BP1, since two adjacent DSBs are not necessarily repaired by the same repair process. The first γH2AX peak appeared to be strikingly similar in width and height to the MRE11 peak, which may be interpreted such that MRE11 was arranged inside the γH2AX-cluster. For 53BP1, the curve, unlike the other proteins, decreased linearly after its peak. This is to be expected because 53BP1 also formed the largest clusters of all proteins and, furthermore, is present in a random distribution off DSB damage areas [49]. The peak of pATM is the highest and broadest one. H3K9me3 is only clustering in small areas, as indicated by the small peak width. However, H3K9me3 also contributes to establishing and maintaining cellular identity. [56]. In principle, it cannot be excluded that the peaks may also be due to the localization accuracy limited by the size of the linker and antibody construct or the distribution and number of epitopes. This fact has been studied elsewhere [81], revealing that these effects have a lower probability than the formation of clusters.

**Figure 4:**
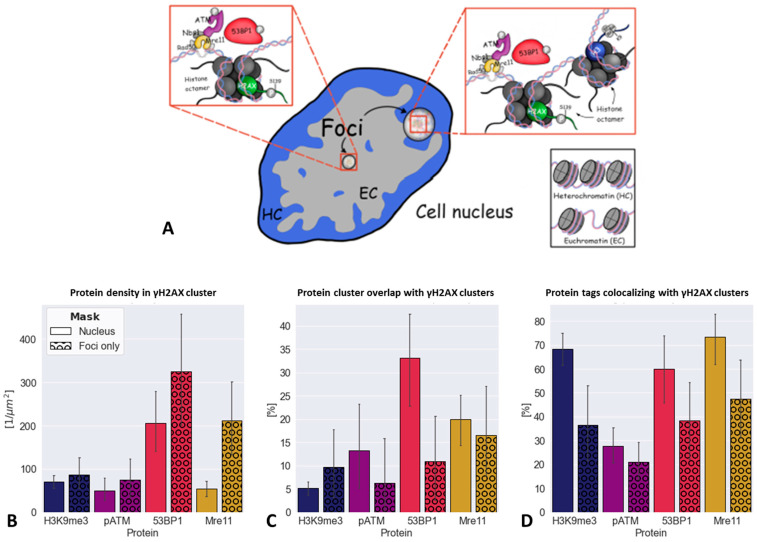
(**A**) Schematic representation of all analyzed DDR factors around DSBs inside repair foci in euchromatin and heterochromatin. (**B**) Protein density within γH2AX clusters for the whole nucleus mask as well as for the masked γH2AX focus areas only. (**C**) Protein cluster overlap (in %) with γH2AX clusters for the entire nucleus and for the γH2AX focus area mask only. (**D**) Number of labeling tags co-localizing with γH2AX cluster areas.

γH2AX occurs around sites of dsDNA breakage as one of the first signals in the DDR and is a relatively precise DSB surrogate marker in low-LET photon irradiation early after irradiation [33,34,38]. For low-LET β-radiation, the number of induced γH2AX foci has been found to correlate with DSB number and can be used as a measure in biological dosimetry [61]. γH2AX foci have been found to co-localize with other DSB repair proteins like 53BP1, MRE11 and pATM. An illustration of the typical repair protein nano-arrangements in a DSB region is shown in Figure 4A. Comparing the density of all repair proteins in γH2AX clusters (Figure 4B), H3K9me3 is very low but not zero, indicating that, in some cases, H3K9me3 tags co-localize with γH2AX tags (see also Figure 4C,D). 53BP1 and MRE11 are mostly concentrated in γH2AX clusters (about 200–300/μm^2^). It is noticeable that a larger area fraction of the 53BP1 clusters overlapped with γH2AX clusters due to the different number and sizes of the marker clusters. Thus, the γH2AX clusters were mostly covered by the 53BP1 clusters, which are similar in size (Figure 4C).

The observed phenomenon that MRE11 forms significantly more clusters as compared to γH2AX (Figure 2) also resulted in a low overlap of γH2AX clusters with MRE11. Thus, MRE11 clusters also existed outside γH2AX foci, off DSB regions (the entire protein MRE11 protein pool was tagged with the antibody used). Randomly distributed MRE11 proteins may have been involved in other processes in addition to DSB repair such as telomere regulation and repair of minor damage [79] or scanning of the genome for dsDNA damage. On the other hand, the proportion of co-localizing MRE11 points within γH2AX clusters with more than 70% reached the highest percentage of signals in the γH2AX clusters. About 60% of 53BP1 co-localized with signals in the γH2AX clusters (Figure 4D). In accordance with the overlay images, H3K9me3 also co-localized with a considerable amount of the γH2AX tag accumulations (Figure 4D). This is contrary to established textbook knowledge, indicating that H3K9me3 heterochromatin is relaxing after irradiation-induced dsDNA damage but the surrounding γH2AX foci signal tags were not intermingling with the heterochromatin tags (see also [35,74]). This reasoned a more detailed analysis of the H3K9me3 organization at γH2AX clustering sites in PBMCs as described below.

The results of co-localizing H3K9me3 and γH2AX elicited the question of whether this is a result of the chosen co-localization limit of 95 nm, i.e., that this value is too large, or whether the repair of DSBs after low-dose β-irradiation might occur within heterochromatin regions in an uncommon way, or whether this is a cell type-specific effect. Since the enrichment of H3K9me3 signals in SMLM at DNA damage sites is thought to indicate de-condensation of heterochromatin (better accessibility for anti-H3K9me3 antibodies), peri-localization of H3K9me3 signal tags with the γH2AX signal tags would suggest that such a re-arrangement of the heterochromatin organization around the DSB sites did not occur [57].

**Figure 5:**
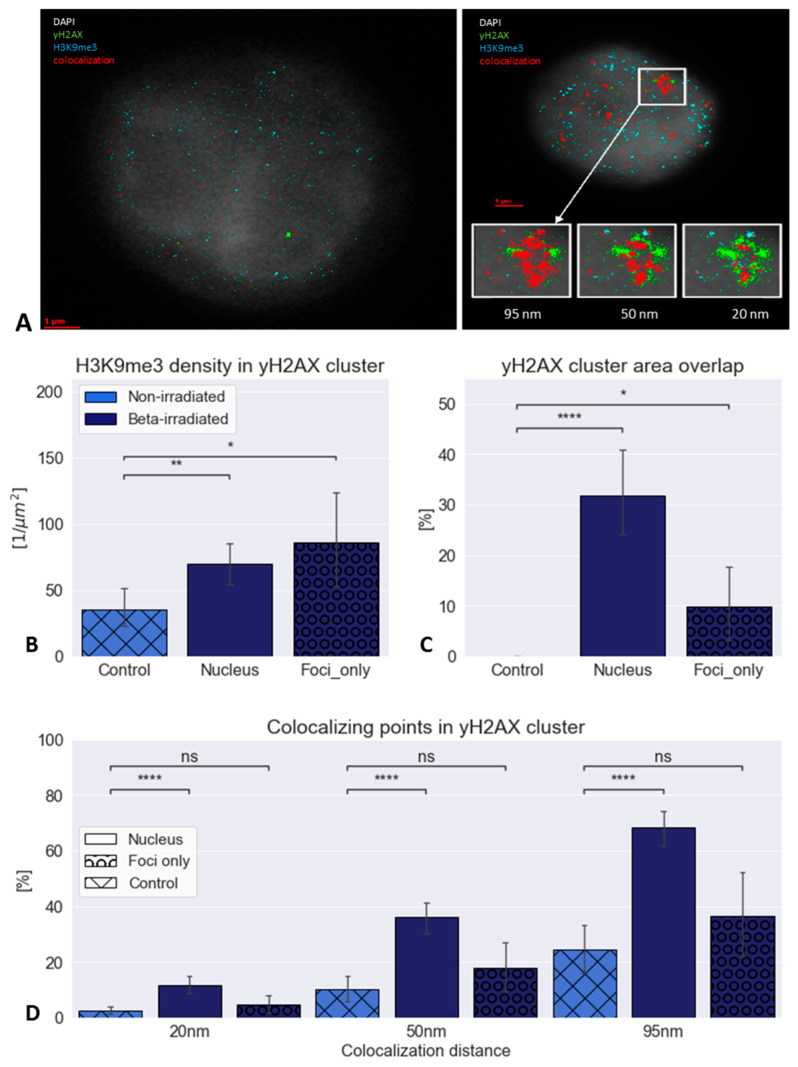
(**A**) Example of an irradiated cell nucleus with SMLM signal tags for γH2AX (green) and H3K9me3 (blue). In the non-irradiated control cell nucleus (left image), only individual co-localizing points (red) are shown that fulfil the 95 nm limit. In the irradiated cell nucleus (left image) a large co-localization cluster is visible. After reduction of the co-localization limit to 50 nm and 20 nm, γH2AX and H3K9me3 co-localization (red signals) remains within the center of the γH2AX cluster even under a distance limit in the range of an antibody size. Scale bar: 1 µm. (**B**) H3K9me3 tag density within γH2AX clusters. (**C**) γH2AX cluster overlap (Note that in the control no γH2AX foci were present and signals represent background staining.) (**D**) Percentage of γH2AX and H3K9me3 co-localization events for the three different distance limits. The increase in the irradiated cell nuclei is significant relative to the control. (Note: For technical reasons γ is written as y). Note: statistical *p*-values: * *p* < 0.05, ** *p* < 0.01, **** *p* < 0.001, n.s. = not significant.

Due to the detected co-localization of H3K9me3 signals with those of γH2AX, the standard threshold value of co-localization (95 nm) was reduced stepwise to 50 nm and even to 20 nm (Figure 5A). Even with the strictest parameter of 20 nm, which is less than the typical fluorophore or antibody size of about 25 nm–30 nm, a considerable co-localization of H3K9me3 and γH2AX tags was found. At the smallest distance, the co-localizing points were surrounded by γH2AX points. The H3K9me3 density was significantly higher within the γH2AX clusters in irradiated cells compared to non-irradiated ones (Figure 5B). In Figure 5C, the γH2AX cluster overlap is shown.

A statistical evaluation (Figure 5D) revealed that the number of co-localizations in irradiated cell nuclei was significantly higher than in non-irradiated control cells for all three co-localization parameters (95 nm, 50 nm, 20 nm), indicating that this was not an accidental effect of chromatin. This significance, however, was not found in the comparison of γH2AX foci with the nucleus control (the area of the nucleus without focal damage). It should be noted that this is not a direct comparison, as the controls showed no foci, and, therefore, no masking was possible, meaning that zero values should be assumed.

**Figure 6:**
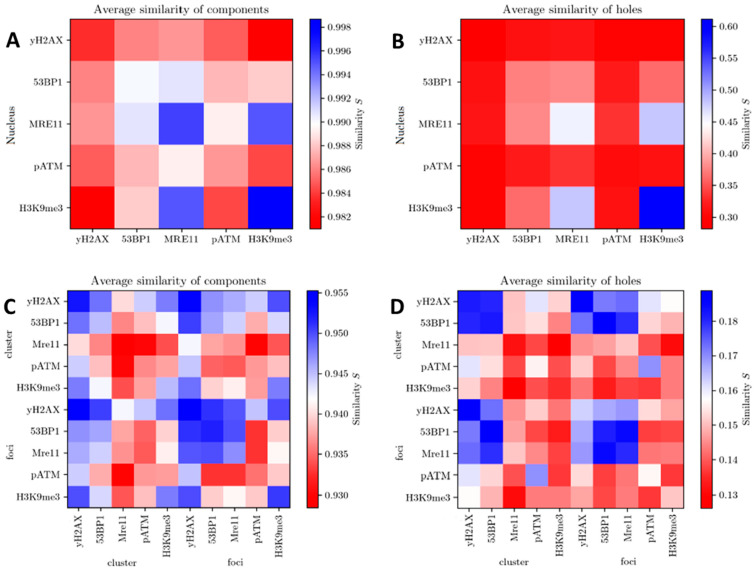
Second-generation heatmaps: Averaged similarities in the nuclei are shown for components (**A**) and holes (**B**). In (**C**,**D**), the respective results are shown for clusters and foci (note: for technical reasons, γH2AX is shown as yH2AX).

Using persistent homology analysis, it is possible to determine the topological properties of signal distributions in clusters or in the entire cell nucleus and to identify recurring structures that are invariant under continuous deformation, i.e., independent of cell orientation, bending or scaling. The alpha-shape method can be used to analyze the geometric scale at which components and holes of point distributions persist. Here, similarities were determined for both parameters (components and holes) using the Jaccard index [75,76]. It should be noted here that the heatmaps of the holes always have a smaller similarity than those of the components, since hole formation only starts at higher α values, while components are already present at α = 0. In addition, there are significantly fewer holes than components in each point distribution of clusters, which reduces the similarity due to the smaller dataset. The similarity values for all labelling sites were calculated, compared (first-generation heatmaps), averaged and presented in second-generation heatmaps (Figure 6).

In Figure 6A,B, the results for irradiated PBMC nuclei are shown. Here, γH2AX clusters are relatively inhomogeneous and compatible with a potential adaptation of the repair process. The same was obtained for pATM signal tag distributions. 53BP1 showed slight similarities to MRE11 and H3K9me3 topology. Figure 6C,D reveal the results for the foci mask and for the DBscan-determined clusters. Here, it is obvious that 53BP1, MRE11 and γH2AX showed strong topological similarity, while pATM did not. The heterochromatin marker H3K9me3 is similarly organized as γH2AX or 53BP1, indicating that the heterochromatin has mostly adapted to the topology of these two tags. This means that the tags of H3K9me3 and γH2AX showed compatible topological arrangements. This may support the results shown in Figure 5.

For the signal distribution within clusters and foci, it is noticeable that the similarity scale decreases not only for components (<0.955) but especially for holes (0.18–0.13). One explanation for this could be the overall lower number of signals when the evaluation was reduced to clusters and foci, which were concentrated to a smaller space with shorter distances between them (α bounded) than within the entire nuclear chromatin space. Holes are presumably more likely to arise for structures that show larger distances. The reduction in hole numbers in clusters and foci might be due to small and, thus, rare underlying structures, which might be more specific for a particular repair protein.

Compared to the signal distribution in the non-damaged nucleus area, a slightly different trend emerged inside the DSB cluster areas: γH2AX and 53BP1 possessed the highest values for S. This is probably due to the fact that both markers had a similar density in their clusters, and 53BP1 was more concentrated in γH2AX clusters than any other protein (highest protein density). In fact, it was shown that similar signal density and a large number of SMLM signal points in the clusters may also lead to increased similarity between the samples [69].

The similarity of the holes is higher when clusters with many substructures are compared that contain characteristic holes. In particular, for the two markers 53BP1 and γH2AX, substructure formation was also observed in the Ripley curves (see Figure 3). The similarity of the nano-organization of DDR proteins in foci showed comparable values to those of the cluster signals, with the exception of MRE11. Here, MRE11 appeared to have higher similarity (higher S-value) within the γH2AX foci than in the detected clusters of the total signal in the nucleus, supporting the observation of additional and topologically different MRE11 clusters not involved in DBS repair (outside γH2AX clusters).

## 4. Discussion

The accumulation of different DDR proteins in so-called foci in the chromatin around Lu-177 β-radiation-induced DSB damage sites was analyzed using SMLM. In contrast to conventional confocal microscopy, this method makes it possible to resolve such repair regions into pointillist arrangements with point distances of down to 10 nm. On this data basis, it was possible not only to precisely localize the position of the individual repair markers investigated but also to apply statistical and topological algorithms for the quantification and comparison of structural organization [23,69]. Since electrons belong to the low-LET particles, low-dose-induced DSBs are produced independently of each other, and usually only one DSB per focus and per electron may be assumed [62]. The beta decay electrons applied here are short ranged. When they induce DNA damage in the nuclear periphery, these electrons may damage heterochromatin in this region. In some cases, larger foci at the border of the nucleus were observed, in which more than one DSB could be present or which may relate to prolonged repair processes.

During Lu-177 decay, electrons with an average energy of 0.13 MeV and energies up to a maximum of 0.5 MeV are released. Stopping power assessments taken from the NIST database helped to give a rough estimate of the relative electron energy (about 10–40%) that is not lost through blood at a corresponding mean path length (CSDA range). This equates to an energy range of around 10–200 keV with which the electrons can penetrate a cell or nucleus until they finally stop and deposit their energy. Monte Carlo simulations [82] showed that for electron energies of 50–200 eV per interaction with DNA, a maximum of 10^−3^ DSB can be generated. This means that DSBs are rare events in the blood samples investigated here. High energies at the top end of the energy distribution curve would lead to typically one DSB per nucleus.

This stands in contrast to our previous investigations using α-particles [23] that belong to the high-LET particulate irradiation. There, Ra-223, an α-emitter with a half-life of 11.43 days and a decay in six steps into stable Pb-207 was used. The progeny Rn-219, Po-215 and Bi-211 are α-emitters as well, so that, in total, four alpha-particles were emitted per Ra-223 decay, depositing energies between 5.77 MeV (Ra-223) and 7.49 MeV (Po-215). These particles cause damage tracks through the chromatin of a cell nucleus. The α-particles studied in this previous publication [23] had a range of up to 90 µm in water and a kinetic energy of about 1.4 to 2.2 MeV per nucleon. They passed cells with a mean LET of about 100 keV/µm. While α-particles lose energy and slow down when traversing matter, their interaction probability increases so that the LET of α-particles peaks towards the end of their tracks at the so-called Bragg peak. The Bragg peak of Ra-223 α particles had an LET of about 220 keV/µm [23]. These principal differences between α- and β-particle irradiation are reflected by the different damage patterns in a cell nucleus, even at similar absorbed doses.

Because of its function as a precise marker of DSBs and direct binding at the DSB surrounding chromatin site, γH2AX tended to form smaller SMLM clusters in contrast to the larger, γH2AX-overlapping domains of 53BP1. This finding is consistent with other investigations, in which cluster size varied depending on the timing of cell fixation after irradiation, i.e., repair time [38,78]. For example, the area of the γH2AX cluster quickly increased with time until all H2AX histones near a DSB were phosphorylated. After successful repair, the cluster size slowly decreased again due to the de-phosphorylation of γH2AX. The signal inside the γH2AX clusters (45%) is also consistent with values reported in previous studies: experiments with γ-irradiated HeLa cells revealed a dose- and time-dependent ratio of 0.1–0.5 of γH2AX SMLM signals inside clusters [35].

In contrast to γH2AX, the protein 53BP1 is always present in the nucleus and able to readily detect DNA damage incurred due to irradiation, which allows for rapid recruitment to the damage site. In addition, 53BP1 formed the largest clusters of all DDR proteins due to its larger size. Furthermore, 53BP1 was the protein with the highest signal tag number inside the DSB clusters relative to γH2AX. Higher signal numbers within protein clusters compared to γH2AX are compatible with other work and have been observed, for example, in fibroblast and brain tumor cells irradiated with heavy ions (Kuentzelmann et al. manuscript submitted). A high level of 53BP1 is expected in terms of radiation-induced DSB damage after low-LET irradiation, since DSBs in eukaryotes are preferentially repaired by NHEJ [83], which strongly depends on 53BP1, as outlined above. Given its role as a molecular scaffold during DSB repair, 53BP1 covers a larger chromatin area [41]. Persistent homology analysis revealed that the signal distribution of 53BP1 in clusters had the highest similarities with γH2AX relative to the other DDR proteins studied.

Like 53BP1, MRE11 occurs in the nucleus also without irradiation. Of all DDR protein samples, the highest signal number and event density were recorded for MRE11 and attributed to the fact that not only the MRE11 molecules involved in DSB repair were labeled but the entire protein pool of MRE11 in the nucleus. This also resulted in more detected clusters (about three times more) than for γH2AX, whose phosphorylation strongly increases after irradiation and is restricted to DSB areas under standard conditions. Thus, in contrast to 53BP1, many protein clusters of MRE11 were also seen outside the outline of γH2AX foci in the localization images. Inside a γH2AX foci mask, the number of MRE11 clusters were similar to those of the γH2AX signal tag values. Hence, some of the tagged MRE11 may be involved in other processes in the nucleus, for instance, telomere regulation and replication-induced damage repair [46,47,79] and/or the detection of the molecules studied failed to reach saturation.

Still, MRE11 clusters were, on average, about 40% smaller than those of γH2AX and were located within γH2AX clusters according to the Ripley distance histograms. However, compared to its distribution in the non-damaged nuclear chromatin, MRE11 formed a larger protein cluster in DSB foci regions. Based on persistent homology comparisons, MRE11 appeared to have higher similarity within the γH2AX foci than in the detected clusters of the total signals throughout the nucleus, supporting the finding of additional and topologically distinct MRE11 clusters not involved in DBS repair (outside a γH2AX focus region). Of all the molecular tags considered, a majority of signals in the γH2AX clusters co-localized most frequently with MRE11. Higher co-localization of γH2AX with MRE11 might correlate with the assumption that MRE11 is involved not only in DSB repair via NHEJ but also via HRR [48]. In our previous publication using Ra-223 α-emitter [23], MRE11 signals were also located in the non-irradiated nuclear environment, suggesting that the damage caused by low-LET β-radiation did not deplete the protein pool. Less than 30% of MRE11 signal tags were inside the γH2AX tracks.

Compared to γH2AX, the active ATM kinase, phosphorylated at S1981, formed a similar number of clusters, but these were approximately 25% smaller (which agrees with other experiments [23]). Of all proteins considered, pATM had the highest signal density within the clusters. According to the distance analysis, pATM seemed to form smaller and more separated (less complex) clusters compared to the other proteins. Moreover, the smaller cluster area also led to a smaller area fraction of γH2AX clusters of pATM, so that pATM had very low co-localization values with γH2AX, in contrast to MRE11 and 53BP1. One reason for this could be a temporally limited association of active and mobile pATM with H2AX histones but not γH2AX in the DSB region. Furthermore, the ATM protein is subject to loss during cell preparation procedures [84].

H3K9me3 is a marker for heterochromatin present in the cell nucleus. H3K9me3 determines the cell type [56]. Here, the largest signal tag clusters were detected due to the high number of signals. However, based on the distance distribution, most of the H3K9me3 signal was randomly distributed in the nucleus at larger distances. Furthermore, persistent homology analysis showed noticeable topological differences in the signal distribution of heterochromatin markers H3K9me3 compared to the DDR proteins. Results obtained after 2 Gy photon irradiation of different cell lines [51,74], where the signal tag cluster regions of γH2AX excluded H3K9me3, showed an increasing distribution of point distances (Ripley statistics) between individual γH2AX and H3K9me3 points. In this case, co-localization of γH2AX and H3K9me3 (95 nm distance criterium) is only possible for border points of these regions. In addition, the visible number of H3K9me3 tags is increasing around γH2AX foci since heterochromatin is usually relaxing [35], so that the accessibility for the anti-H3K9me3 antibody is improved. Here, however, more than 70% of H3K9me3 tags were inside the γH2AX foci, and about 40% showed co-localization. Moreover, co-localization was also found with the strictest parameter (20 nm < fluorophore size of about 30 nm). The close spacing of the two H3K9me3 and γH2AX histone marks in the DSB region of irradiated cells suggests that γH2AX forms at or inside human heterochromatin regions. It may also be that compacted heterochromatin remains in the direct neighborhood of phosphorylated histone H2AX chromatin that is maintained in G1 cells, while repair may be postponed to cell cycle progression after irradiation in the low mGy range [85]. In the latter article, the authors observed γH2AX DSB foci that persisted after low-LET irradiation below 200 mGy but were quickly repaired after cell cycle re-entry or pre-treatment with oxygen radicals. This suggests that some γH2AX foci in heterochromatin regions may not be repaired at low doses and maintained for later repair. However, since the current irradiation lasted only for 1 h, the latter possibility requires further investigation.

## 5. Conclusions

SMLM and sophisticated mathematical approaches of Ripley distance frequency statistics and persistent homology were successfully applied to analyze the spatial arrangements of damage sites, repair proteins and surrounding heterochromatin distribution at DSB-containing chromatin areas after in-solution irradiation with the β-emitter Lu-177. The obtained data suggest that all these damage sites follow characteristic rules of spatial organization. Based on protein densities, 53BP1 was most abundant at DSB sites after β-irradiation, indicating the occurrence of, compared to α-irradiation, less complex damage and the preference for fast repair by NHEJ. However, larger γH2AX foci were also observed at the periphery of the nuclei. Combined with the co-localization of γH2AX clusters with H3K9me3 signals, this suggests either the appearance of some rarer and more complex DSBs in the perinuclear heterochromatin region or a persistence of DSB damage in heterochromatin until later repair.

## Data Availability

The primary datasets and the evaluation software are part of the KIP SMLM data archive and can be obtained upon request, including necessary support in data handling, from the corresponding author, M.H.
